# Association of Aerobic Fitness With Mnemonic Discrimination and Generalization in Older Adults

**DOI:** 10.1093/geroni/igaf122.3225

**Published:** 2025-12-31

**Authors:** Jeongwoon Kim, Bryan Montero Herrera, Chadsley Wessinger, Kylie Kayser, Brittany Armstrong, Christopher Wahlheim, Jennifer Etnier, Kyoung Shin Park

**Affiliations:** Emory University School of Medicine, Atlanta, Georgia, United States; University of North Carolina at Greensboro, Greensboro, North Carolina, United States; University of North Carolina at Greensboro, Greensboro, North Carolina, United States; The University of North Carolina at Greensboro, Greensboro, North Carolina, United States; University of North Carolina at Greensboro, Greensboro, North Carolina, United States; University of North Carolina at Greensboro, Greensboro, North Carolina, United States; University of North Carolina at Greensboro, Greensboro, North Carolina, United States; Emory University School of Medicine, Atlanta, Georgia, United States

## Abstract

The hippocampus is responsible for learning and memory and vulnerable to age-related decline. Mnemonic discrimination (MD) and mnemonic generalization (MG) are sensitive markers of hippocampal integrity. MD reflects the ability to successfully separate similar information while MG reflects an increasing overlap when encoding similar information. A growing body of research suggests that aerobic fitness (AF) may protect hippocampal integrity in older adults. However, the extent to which MD and MG are associated with AF remains underexplored. In this preliminary study, we examined cross-sectional associations between AF with MG and MD in 60 cognitively unimpaired, ambulatory older adults (mean age 70.6±4.6 years; 86.7% female). We hypothesized negative and positive associations of AF with MG and MD, respectively. AF was assessed through the total distance walked in 6 minutes (6MWD). MG and MD were assessed through the Mnemonic Similarity Task and operationalized as the old (incorrect) and similar (correct) categorization of lure items, respectively. The hypothesis was tested through hierarchical regressions with covariates (age, sex, and education) in step 1 and 6MWD in step 2. Upon adjusting for covariates, 6MWD was inversely associated with MG (β=-0.30, p = 0.041) and had a trend-level positive association with MD (β = 0.25, p = 0.060). These findings indicate that older adults with higher AF may better separate and store information in a non-overlapping manner, implying protective effects of AF on hippocampal-dependent mnemonic discrimination in cognitive aging. Enhancing AF may represent a promising strategy to protect hippocampal integrity among the growing aging population.

